# Impact of Reconstructing Intercostal Artery on Spinal Cord Circulation During Open Surgery for Thoracoabdominal Aortic Aneurysm

**DOI:** 10.21470/1678-9741-2021-0219

**Published:** 2023

**Authors:** Kei Kobayashi, Satoshi Saito

**Affiliations:** 1 Department of Cardiovascular Surgery, Heart Institute of Japan, Tokyo Women’s Medical University, Tokyo, Japan.

**Keywords:** Aortic Aneurysm, Thoracic, Arteries, Cerebrospinal Fluid Leak, Evoked Potentials, Motor, General Surgery, Hospitals, Hypothermia, Spinal Cord Injuries, Mortality

## Abstract

**Introduction:**

We evaluated the outcomes of the selective intercostal artery reconstruction for preventing spinal cord injury during thoracoabdominal aortic aneurysm repair.

**Methods:**

We retrospectively assessed 84 consecutive patients who underwent thoracoabdominal aortic aneurysm repairs between 2004 and 2016. The mean age of the patients was 57.3 years. We performed preoperative multidetector computed tomography in 74 patients (88.0%) to identify the Adamkiewicz artery. Spinal cord injury preventive measures included motor evoked potential monitoring, hypothermia induction, Adamkiewicz artery or other intercostal artery reconstruction, and cerebrospinal fluid drainage.

**Results:**

The hospital death rate was 5.9%, and paraplegia occurred in four patients (4.7%). The Adamkiewicz artery or other intercostal arteries were reconstructed selectively in 46 patients (54.7%). Of these patients, 41 underwent postoperative multidetector computed tomography, which revealed occlusion of the reconstructed grafts in 23 patients (56.0%). There was no paraplegia in the patients who underwent reconstruction of the Adamkiewicz artery, which was patent on postoperative multidetector computed tomography. Univariate analysis showed no significant effect of various risk factors on the development of spinal cord injury.

**Conclusion:**

Outcome of open surgery for thoracoabdominal aortic aneurysm in our institution regarding spinal cord injury was satisfactory. The benefits of Adamkiewicz artery reconstruction remain inconclusive, and further larger studies are required to identify its validation for spinal cord protection in thoracoabdominal aortic aneurysm repair.

## INTRODUCTION

Spinal cord injury (SCI) remains one of the most catastrophic complications following open surgery for thoracoabdominal artery aneurysm (TAAA) repair^[1]^. Although the incidence of intractable neurologic complications has declined owing to advances in anesthetic and surgical techniques, the rate of paraplegia and paraparesis still ranges from 5% to 15%^[1,2,3,4,5]^.

For spinal cord protection, various methods have improved the surgical outcomes. These methods include distal aortic perfusion^[6]^, hypothermia induction^[7]^, motor evoked potential (MEP) monitoring^[3,8,9]^, preservation or reattachment of the responsible intercostal arteries (ICA)^[4,10,11]^, and cerebrospinal fluid drainage (CSFD)^[12]^. Although the causes of SCI in aortic surgeries are considered multifactorial, a more accurate comprehension of the spinal cord circulation can provide important information crucial for preventing SCI.

In the thoracolumbar region, the great anterior medullary artery (*i.e.*, the Adamkiewicz artery [AKA]) is the dominant feeder of the spinal cord. Magnetic resonance angiography^[13,14]^ and multidetector computed tomography (MDCT)^[15,16]^ have been reported to be useful for noninvasive AKA detection. With these relatively new technologies, preoperative AKA detection is possible and is very useful for reducing the incidence of ischemic injury of the spinal cord^[16,17]^.

Currently, there are considerable numbers of reports on how to effectively reconstruct AKA and other ICA during TAAA repair to prevent SCI^[18,19]^. We therefore retrospectively investigated the outcomes of reconstruction of ICA during open surgery for TAAA repair at our institution, focusing mainly on their patency, cord perfusion, and SCI prevention. This study presents a descriptive cohort of patients who underwent to thoracoabdominal aneurysm reconstruction and aims to evaluate the ICA reconstruction impact on patency, spinal cord perfusion, and injury prevention.

## METHODS

### Study Design

This is a retrospective study of patients who underwent open surgery TAAA repairs at Tokyo Women’s Medical University Hospital between January 2004 and September 2016. This study was approved by the ethics committee of our institute (approval number: 5467). Medical records were reviewed, and the following data were retrieved and analyzed: basic demographic data, anatomical information, surgical history, intraoperative data, and postoperative outcomes.

### Repair Strategies

MDCT for AKA identification was routinely performed for all patients whenever possible, even in urgent settings. The criteria for AKA detection were as follows: (1) artery being continuous to the AKA with a hairpin turn on an early phase image, (2) extension of the vessel to the anterior midsagittal surface of the spinal cord from the radicular-medullary artery originating from the dorsal branch of the intercostal or lumbar artery, and (3) the signal intensity in the early phase diminishes in the late phase.

All operations were performed in the right oblique position. The skin incision was done along the anterior axillary line extended to the left pararectal line. AKA was reconstructed unless it was not identified, it was occluded, or it was very small. All operations were performed under either mild hypothermia (34ºC) on partial cardiopulmonary bypass with perfusion of ICA, or deep hypothermic circulatory arrest (DHCA) (18ºC), depending on whether clamping of the proximal aorta was possible or not. During aortic cross-clamping, distal aortic perfusion at > 80 mmHg was maintained by partial cardiopulmonary bypass consisting of a heparin-coated femorofemoral circuit, with permissive mild hypothermia at 32°C to 34°C. Anastomoses were performed using a “segmental clamp technique”^[11]^ to reduce the time of spinal ischemia. In cases with visceral arterial involvement, visceral perfusion was also performed using 12F or 14F branched balloon-tipped tubes of the cardiopulmonary bypass circuit. In DHCA circumstances, distal anastomosis and reconstruction of the visceral arteries and ICA were carried out during rewarming. In addition, the distal perfusion flow was increased to raise the mean distal pressure > 80 mmHg. The blood pressure of the upper body was also increased using catecholamines, transfusion, or both. MEP monitoring was introduced in all operations. MDCT was performed before discharge.

### Cerebrospinal Fluid Drainage

CSFD was performed in patients in whom anticoagulant drugs or DHCA was not used to avoid epidural hemorrhage. Cerebrospinal fluid was allowed to freely drain with gravity whenever the cerebrospinal fluid pressure was > 10 mmHg. In patients without a spinal cord deficit, the drain was removed on postoperative day two.

### Motor Evoked Potentials

For physiological assessment, MEPs were also monitored during surgery^[3,8]^. Under adequate anesthesia with low doses of fentanyl (0.02-4 mg/kg), propofol (4-6 mg.kg–1.h–1), and vecuronium (0.04 mg.kg–1.h–1), the motor cortex was activated by transcranial electrical stimulation at 600 V. The action potentials conducted through the anterior horn motor neurons were recorded from the skin over the upper extremity muscles (as a control), the lower extremity muscles, and the thenar muscle. Light anesthesia was maintained with a small dose of fentanyl and propofol. During cross-clamping, or during cardiac arrest, MEP levels were determined every fve minutes. A fall in the MEP amplitude to < 25% of the baseline was taken to indicate as ischemia of the spinal cord^[8]^.

### Statistical Analysis

The statistical analysis was carried out using JMP14 (SAS Institute, Cary, North Carolina, United States of America). Continuous variables are expressed as mean ± standard deviation, and categorical variables are expressed as number of patients. Variables were compared between groups using logistic regression analysis. A *P*-value of < 0.05 was considered to indicate a statistically significant difference.

## RESULTS

### Patient Demographics

Eighty-four consecutive patients who underwent open surgery TAAA repairs were included in this study. The demographic data and associated conditions of the patients are summarized in Table 1. There were 27 women (32.1%) and 57 men (67.9%), and 23 patients had Marfan syndrome (27.3%). Their mean age was 57.3 years. All aneurysms limited to the thoracic aorta (ascending, arch, or descending) were excluded. This left 25 type I (29.7%), 34 type II (40.4%), 22 type III (26.1%), and three type IV (3.8%) aneurysms according to the Crawford classification. In the Stanford classification, there were 10 type A aortic dissections (11.9%), 40 type B aortic dissections (47.6%), 31 patients with no dissection (36.9%), and three with pseudoaneurysm (3.6%). The mean minimum diameter of the descending artery was 60.4±16.4 mm.

**Table 1:** Clinical data.

Variable	
Total number of patients	84
Male sex, n (%)	57 (67.8)
Marfan syndrome, n (%)	23 (27.3)
Age, years, mean ± SD	57.3±15.1
Size of aneurysm, mm, mean ± SD	60.4±6.4
Diabetes, n (%)	4 (4.7)
Hypertension, n (%)	77 (91.6)
Dyslipidemia, n (%)	21 (25.0)
Coronary artery disease, n (%)	13 (15.4)
Renal failure, n (%)	20 (23.8)
Dialysis, n (%)	4 (4.7)
Cerebrovascular disease, n (%)	14 (16.6)
Aortic pathology, true aneurysm, Crawford classification	
Type I, n (%)	25 (29.7)
Type II, n (%)	34 (40.4)
Type III, n (%)	22 (26.1)
Type IV, n (%)	3 (3.8)
Aortic dissection, Stanford classification	
Type A, n (%)	10 (11.9)
Type B, n (%)	40 (47.6)
Pseudoaneurysm, n (%)	3 (3.6)
Preoperative AKA identification, n (%)	74 (88.0)

AKA=Adamkiewicz artery; SD=standard deviation

### Preoperative Multidetector Computed Tomography Study

**Fig. 1:**
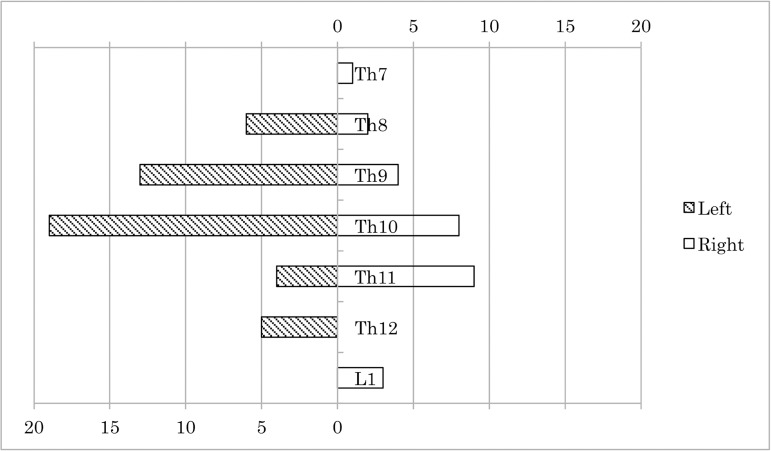
Distribution of the location of the Adamkiewicz artery.

Of the 84 patients assessed, we identified AKA in 74 patients (88.0%). The laterality and distribution of the AKA locations are shown in Figure 1. AKA originated predominantly from the left intercostal or lumbar arteries in 55.9% of the cases.

### Surgical Outcome

The operative details are shown in Table 2. There were 31 patients (36.9%) who underwent operation under DHCA. AKA was reconstructed in 39 patients (46.4%), preserved in 26 patients (30.9%), sacrificed in seven patients with their other ICA reconstructed (8.3%), and sacrificed in 12 patients with their ICA not reconstructed (14.4%).

**Table 2:** Operative details.

Variable	
Operative time, min, mean ± SD	577.7±150.4
Cardiopulmonary bypass time, min, mean ± SD	192.4±85.8
Aortic cross-clamping time, min, mean ± SD	139.6±55.8
Bleeding, ml	3435.9 (318-17090)
Transfusion, ml	7558.4 (1300-28070)
DHCA, n (%)	31 (36.9)
Circulatory arrest time, min	25.7
AKA	
Reconstruction of AKA, n	39
Preservation of AKA, n	26
Sacrifice of AKA, no reconstruction of other ICAs, n	12
Reconstruction of other ICAs, n	7
MEP	
No change, n	35
Transient decrease, n	44
Disappeared, n	2
Unidentified, n	3

AKA=Adamkiewicz artery; DHCA=deep hypothermic circulatory arrest; ICA=intercostal artery; MEP=motor evoked potential; SD=standard deviation

The MEP showed no changes in 35 patients (41.6%), decreased transiently in 44 patients (52.3%), and disappeared in two patients (2.3%). Changes in the MEP could not be identified in three patients.

**Fig. 2:**
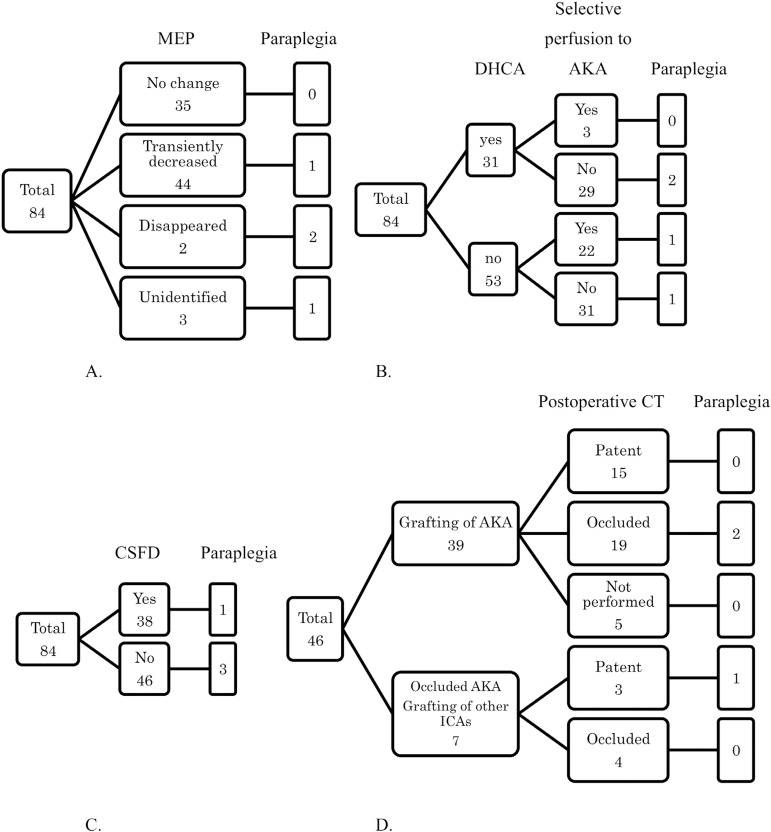
Relations between paraplegia and spinal cord protection measures. A) Changes in motor evoked potential (MEP) during operation; B) use of deep hypothermic circulatory arrest (DHCA) and selective perfusion to Adamkiewicz artery (AKA); C) use of cerebrospinal fluid drainage (CSFD); D) intercostal artery (ICA) reconstruction. CT=computed tomography.

There was no intraoperative death in any patient. The hospital death rate was 5.9%, and paraplegia occurred in four patients (4.7%). Among the patients who had reconstructed AKA or other ICA (n=46), 41 patients (89%) underwent postoperative MDCT. The interposed graft was patent in 18 patients (44%) and occluded in 23 patients (56%) as confirmed by MDCT postoperatively. Figures 2 A-D shows the relations between paraplegia and CSFD, DHCA, ICA reconstruction, and MEP monitoring.

Paraplegia was not evident in patients who underwent reconstruction of AKA, which was patent on postoperative MDCT. On the other hand, one of three patients who underwent reconstruction of ICA other than the AKA had paraplegia even if the ICA was patent on postoperative MDCT.

### Risk Factor Analysis for SCI

Univariate analysis of the risk factors showed no significant risk factors for SCI prevention. Cardiac pulmonary bypass time, DHCA use, and ICA reconstruction were not significant risk factors. Univariate analyses of the risk factors are summarized in Table 3.

**Table 3:** Univariate analysis of risk factors for spinal cord injury.

Variable	Odds ratio	95% CI	*P*-value
Male sex	-	-	0.15
Age > 65 years	0.45	0.04-4.52	0.48
Hypertension	-	-	0.53
Dyslipidemia	1.00	0.09-10.16	1.00
Diabetes mellitus	-	-	0.64
Renal failure	1.07	0.10-10.90	0.95
Smoking	3.00	0.39-22.71	0.26
Marfan	-	-	0.15
Size of aneurysm	1.02	0.98-1.06	0.20
Dissection	2.10	0.20-21.1	0.51
Operative time	1.00	0.99-1.00	0.75
Cardiopulmonary bypass time	1.00	0.99-1.01	0.52
Aortic cross-clamping time	1.00	0.99-1.03	0.30
Bleeding	1.00	0.99-1.00	0.31
CSFD	0.38	0.03-3.88	0.40
DHCA	2.2	0.29-16.5	0.43
Reconstruction or preserved ICA	0.47	0.04-5.01	0.53
Selective perfusion	0.77	0.07-7.86	0.83

CI=confidence interval; CSFD=cerebrospinal fluid drainage; DHCA=deep hypothermic circulatory arrest; ICA=intercostal artery

### Details of Patients in Paraplegia

Table 4 shows the details of patients in paraplegia (n=4). In the first case, AKA was occluded preoperatively, and the other ICA were reconstructed. An episode of hypotension occurred intraoperatively, and the patient became paraplegic in spite of the patent interposed graft.

**Table 4:** Details of paraplegia in patients.

	Age (years)	Sex	Marfan	Types of aneurysm/dissection	CSFD	MEP	DHCA	Selective perfusion	AKA	Postoperative MDCT
1	62	Male	x	Stanford type B, Crawford (I)	**○**	Not performed	x	x	Occluded, grafting of other ICAs	Patent
2	78	Male	x	Pseudoaneurysm, Crawford (III)	x	Disappeared	x	○	Occluded, no grafting	N/A
3	55	Male	x	Stanford type B, Crawford (III)	x	Transient	○	x	Bypass grafting	Occluded
4	59	Male	x	Stanford type B, Crawford (I)	x	Disappeared	○	x	Bypass grafting	Occluded

AKA=Adamkiewicz artery; CSFD=cerebrospinal fluid drainage; DHCA=deep hypothermic circulatory arrest; ICAs=intercostal arteries; MDCT=multidetector computed tomography; MEP=motor evoked potential

The second case involved a patient with infectious pseudoaneurysm. The patient’s general status was very poor to perform DHCA. The perivascular tissue was very adhesive, which made it difficult to clamp the aorta and resulted in excessive bleeding. The MEP disappeared during the operation.

The third case had a history of renal transplantation and ongoing hemodialysis. The patient became paraplegic after developing hemorrhagic shock postoperatively. The reconstructed AKA was occluded on postoperative MDCT.

In the fourth case, the AKA was reconstructed. However, during hemostasis, the MEP disappeared. The interposed graft was occluded on postoperative MDCT.

## DISCUSSION

### Effects of Selective Reconstruction of Adamkiewicz Artery on Surgical Outcome

The obtained information on the blood supply to the spinal cord provides a “map” of the relevant intercostal or lumbar arteries suitable for reconstruction or preservation. Preoperative knowledge of the arteries requiring reconstruction or preservation is highly advantageous. Moreover, the preoperative identification of the AKA aids in the preoperative and intraoperative surgical planning. The safest segmental cross-clamp site can easily be determined as the target vessels to be vascularized based on the preoperative anatomical assessment of the AKA. Kawaharada et al.^[13,14]^ have also reported on the usefulness of the preoperative identification of the AKA by magnetic resonance angiography. However, the preoperative demonstration of the AKA cannot prevent all of SCIs.

The combined use of preoperative AKA identification and MEP monitoring has been reported to be important for preventing neurological deficit^[3]^. MEP monitoring is, however, affected by peripheral ischemia, anesthesia including neuromuscular blockade, and systemic hypothermia.

Our adjunctive spinal cord protection measures might have contributed to the low incidence of neurological deficits. CSFD has been demonstrated by several prospective studies to significantly enhance spinal cord protection^[20]^. It is used by many surgeons intraoperatively and postoperatively to minimize resistance to blood inflow into the intrathecal space. Griepp et al.^[21]^ reported how the occurrence of steal into the distal circulation can be prevented by distal perfusion. This involves maintaining a higher mean arterial pressure of 80 mmHg at the minimum and 90-100 mmHg at the maximum during the operative procedure. We suggest that the combined use of these modalities is superior to their individual use.

### Effects of Selective Reconstruction of Adamkiewicz Artery on Postoperative Spinal Cord Circulation

The comparison between the preoperative and the postoperative MDCT showed variable information regarding the changes in spinal cord circulation during the perioperative phase. Among all the patients evaluated, 46 patients could not have AKA preservation, 39 patients (84.7%) underwent AKA reconstruction, and seven patients underwent other ICA reconstruction under the guidance of the preoperative MDCT study. Although four patients developed paraplegia, this condition was not observed in patients who underwent reconstruction of the AKA, which was patent in the postoperative MDCT study. On the other hand, one patient whose AKA was occluded and whose other ICA were reconstructed developed paraplegia despite the patent ICA graft in the postoperative MDCT study. Surprisingly, 23 patients had occlusion of their grafts for AKA or other ICA.

Tanaka et al.^[22]^ reported the positive efficacy of preoperative AKA identification and reconstruction or preservation of AKA for spinal cord protection. Our results are compatible with the report. However, our findings also suggests that, in some patients, postoperative AKA patency is important for maintaining the overall blood flow to the spinal cord, whereas in other patients, postoperative ICA or AKA occlusion does not lead to spinal cord ischemia. The blood flow to the spinal cord varies among diferent individuals. Furthermore, this blood flow will be altered after surgery for abdominal or thoracic aortic aneurysm with the development of collateral blood flow.

The occlusions of the interposed graft targeted to the AKA or other ICA can result from technical failure and the development of collaterals during the perioperative phase. Technical difficulties are undoubtedly involved when anastomosing the Dacron graft to the fragile aortic wall that surrounds the ostia of the ICA. It is also possible that even a temporal blood supply via the interposed graft for spinal cord perfusion facilitates the development of collaterals during the early postoperative period. This could allow the development of major collaterals. Christian et al.^[23]^ reported that within fve days after ICA are occluded, profound anatomical alterations in the intraspinal and paraspinous arteries and arterioles occur, providing the anatomical substrate for preservation of spinal cord blood flow via collateral pathways in pigs. It is suggested that patency of reconstructed ICA during the early postoperative period plays an important role in prevention of SCI.

There exist axial networks of small arteries in the spinal canal (*i.e.*, perivertebral tissues) and paraspinous muscles that anastomose with one another, as well as nutrient arteries in the spinal cord^[21,24]^. Okita et al.^[25]^ reported that post-bypass hypotension is one of the risk factors of developing SCI. Bleeding from ICA during open surgery can also contribute to a reduction in the blood supply to the spinal cord. All four of the cases that developed paraplegia had some unstable hemodynamics during surgery or on the postoperative period. Although there were no significant differences in prevention of SCI, unstable hemodynamics at perioperative period are possibly attributed as one of the risk factors.

There were 10 (12%) patients with unidentified AKA preoperatively. Those patients include either obstructed AKA or no AKA. There was no paraplegia among these patients despite only three patients (30%) reconstructed ICA according to the intraoperative decision. This suggests that some development of collateral blood flow was already made preoperatively.

Although some positive effects were suggested in other reports, the risk factor analysis did not show the considerable validative effects of AKA grafting. Considering the dissociation of the lower clinical paraplegia incidence and the higher rate of AKA graft occlusions postoperatively, the effects of AKA grafting on SCI prevention can not to be defined as in other studies. However, patency at early postoperative phase may contribute to low rate of paraplegia, and reporting the fact of intercostal graft occlusion should be consider in the future surgery. It is evident that further larger clinical studies are necessary to validate the impact of selective reconstruction of the preoperatively identified AKA during TAAA repair.

### Limitations

This study is a non-randomized, observational, retrospective review of prospectively collected data involving a relatively small cohort of patients. This study is a descriptive experience of a given operation and not to bring up the idea of a comparative study of the impact of AKA reconstruction against any reconstruction in the prevention of SCI. Moreover, 10.8% of the patients could not undergo postoperative MDCT because of either early mortality or renal dysfunction.

## CONCLUSION

The selective reconstruction of the preoperatively identified AKA during TAAA repair is safe and clinically effective “for preventing SCI” when combined with adjunctive measures. The benefits of AKA reconstruction remain inconclusive, and further larger studies are required to identify its validation for spinal cord protection in TAAA repair. Therefore, careful consideration of this collateral source for the spinal cord as well as optimal imaging for guided segmental arterial reconstruction are important.
